# Characterization and Pb(II) removal potential of corn straw- and municipal sludge-derived biochars

**DOI:** 10.1098/rsos.170402

**Published:** 2017-09-20

**Authors:** Shujuan Wang, Wei Guo, Fan Gao, Rui Yang

**Affiliations:** 1 School of Environmental Science and Engineering, North China Electric Power University, Beijing 102206, People's Republic of China; 2 Beijing Key Laboratory of New Technique in Agricultural Application, Beijing, People's Republic of China

**Keywords:** adsorption, lead, biochar, sewage sludge, pyrolysis

## Abstract

Corn straw- and municipal sludge-derived biochars (CS-BC and MS-BC, respectively) were used to remove Pb(II) from aqueous solutions. Despite being pyrolysed at the same temperature (723 K), MS-BC showed higher porosity and hydrophobicity than CS-BC. The optimum biochar loading and pH values allowing efficient Pb(II) removal (greater than 80%) were 0.2 g l^−1^ and 7.0, respectively. The presence of PO_4_^3−^ (greater than 0.01 mol l^−1^) significantly affected the adsorptive performance of Pb(II) on the biochar samples. The adsorption data fitted well to a pseudo-second-order kinetic model and a Langmuir model, and the maximum Pb(II) adsorption capacities were 352 and 387 mg g^−1^ for CS-BC and MS-BC, respectively. The main mechanisms involved in the adsorption of Pb(II) on biochar were electrostatic attraction and surface complexation. When comparing both biochars, CS-BC showed better cost-effectiveness for the removal of Pb(II) from aqueous solutions.

## 1. Introduction

The development of the Chinese economy and society in recent decades has significantly improved the living condition of the people. However, the living environment, particularly water and soil, are being seriously deteriorated by heavy metal due to the discharge from industry and urbanization construction [1]. Lead (Pb) is a well-known toxic heavy metal typically used as a raw material in petrochemical, printing, battery, pigment and photographic material applications, among others [2,3]. Pb can increase the health risk of ecosystems and organs at low concentrations (ng l^−1^) as it accumulates in the nerve, blood, kidneys and immune system [4]. Hence, this toxic metal should be effectively removed from aqueous environments in order to protect human health and ensure ecological safety [5].

Several techniques are currently used to remove heavy metals from aqueous solutions including adsorption, chemical precipitation, oxidation/reduction, ion-exchange, coagulation/flocculation and membrane filtration [6]. When considering efficiency, effectiveness, technical flexibility and economic feasibility, adsorption is the most promising method among these techniques [7]. Adsorption is an ion sequestration process via physi- and/or chemisorption, complexation and ion-exchange phenomena [8]. In recent years, a great diversity of living or non-living biomass materials have been used as sorbents to remove heavy metals from aquatic environments and to trap CO_2_ in soil [9,10]. Among these sorbents, biochar is preferred owing to its easy handling, wide availability of raw materials and inexpensiveness characteristics.

Biochar is a carbon-rich solid produced by pyrolysis of biomass such as wood and agricultural wastes [11]. Biochars can be used as sorbents for the removal of heavy metals (e.g. Pb, Cd, Cr, Co and As) from water solutions [12–14]. In this sense, sugar cane bagasse- and orange peel-derived biochars reached Pb(II) removal capacities as high as 87.0 mg g^−1^ (pH = 9.6, 298 K), and the presence of carboxyl, hydroxyl and carbonyl groups on the biochar material was mainly responsible for the adsorption of Pb(II) [3]. The high positive surface charge of biochar under acidic environments hinders the adsorption of Pb(II) ions via electrostatic repulsion, whereas Pb(II) easily precipitated as hydroxide under alkaline conditions [11]. Moreover, some anions present in aquatic environment such as Cl^−^ and ${\mathrm{NO}}_{3}^{-}$ can interact with heavy metals (e.g. Cd, Zn and Cu) thereby affecting their adsorption capacity and removal efficiency [15]. The different surface functional groups, internal voids and surface charge characteristics of biochar materials as a result of the variability of biomass sources and pyrolytic temperatures can complicate the adsorption mechanisms and the behaviour of heavy metals on biochar [11,16].

Rapid population growth and urbanization have resulted in a higher production of agricultural and urban wastes (e.g. corn straw and municipal sludge, respectively), especially in developing countries. For example, the annual production of corn straw and dry municipal sludge in China reached 0.26 billion tons and 6.25 million tons, respectively [17,18]. Therefore, the conversion of these wastes into biochar sorbents is a ‘win–win’ solution for improving waste treatment while removing heavy metals from aquatic environments. However, the adsorption mechanism and behaviour of Pb(II) on corn straw- and municipal sludge-derived biochars in aqueous solutions under different influence factors (especially coexistence of anions such as PO_4_^3−^ and ${\mathrm{CO}}_{3}^{2-}$) have been scarcely treated in the literature. Additionally, the adsorption mechanism of Pb(II) on these biochars remains partly unknown. To fill this gap, we used corn straw- and municipal sludge-derived biochars (CS-BC and MS-BC, respectively) to remove Pb(II) from aqueous solutions. The aims of the present work were (i) to determine the adsorption capacity of Pb(II) over CS-BC and MS-BC in aqueous solution; (ii) to discuss the influence of biochar loading, pH, coexisting anions, reaction time and temperature on the Pb(II) adsorption process; (iii) to compare and analyse the potential of these biochars for removing Pb(II).

## 2. Material and methods

### 2.1. Materials

The biochars were prepared in the laboratory according to the method described elsewhere [11]. The pyrolysis temperature to produce biochar was 723 K based on the previous research that a relatively high temperature (673–773 K) can produce the well-carbonized biochar with higher surface area and porosity which can quickly and effectively sorb the pollutants [19,20]. Two different types of biomass (i.e. corn (maize) straw and sludge) were collected from the test field of the Beijing University of Agriculture (40.22° N, 116.23° E) and Gaobeidian Sewage Treatment Plant in Beijing (36.68° N, 115.78° E), respectively. The corn straw raw materials were washed three times with ultrapure water to remove the impurities. Corn straws were subsequently dried at 333 K for 24 h and finally, pyrolysed at 723 K for 2 h in a ceramic fibre furnace (TC-2.5-10, Beijing ZhongXing WeiYe Instrument Co., Beijing, China) under oxygen-limited conditions to produce biochar. Owing to the domestic origin of municipal sludge, the amounts of heavy metals in raw material and in the produced biochar were relatively lower based on the previous study [21] and did not exceed the EPA thresholds for land application of sewage sludge [22]. Moreover, pyrolysis at this temperature can effectively immobilize some toxic matter (such as heavy metals) in sludge to reduce bioavailability and improve the safety during reuse of the sludge [23]. The collected municipal sludge samples were dried at room temperature, sieved (100-mesh), and finally pyrolysed at 723 K for 2 h to produce biochar. After natural cooling, the produced biochars (CS-BC and MS-BC) were stored in brown glass bottles. Analytical reagent grade chemicals and ultrapure water were used throughout this study. A Pb(II) stock solution (1000 mg l^−1^) was prepared by dissolving Pb(NO_3_)_2_ in ultrapure water. The pH of the experimental solutions was adjusted using 0.1 mol l^−1^ NaOH or HNO_3_ solutions. NaNO_3_, Na_2_CO_3_ and Na_3_PO_4_ stock solutions (1 mol l^−1^) were prepared by dissolving the corresponding amount of the chemicals in ultrapure water.

### 2.2. Characterization of biochars

The as-prepared CS-BC and MS-BC samples were characterized by elemental analysis (Vario EL, German Elementar Co., Germany), scanning electron microscopy (SEM, S250MK3, Cambridge UK Co., UK), Fourier transform infrared spectroscopy (FTIR, Germany BRUKER Spectrometer Co., Germany) and X-ray diffraction (XRD, X'Pert PRO MPD, Holland Research Co., The Netherlands). Elemental analysis was used to determine the C, H, N, O and S contents of the two different biochars. The Brunauer–Emmett–Teller (BET) surface areas and the micropore volumes (MV) were determined from N_2_ adsorption isotherm data obtained at 77 K on an accelerated surface area and porosimetry system (ASAP 2020, Micromeritics, USA). SEM was used to determine the structure of the two different biochars. XRD analysis of two different biochars was carried out on a diffractometer provided with Cu Kα radiation (Kα = 1.54 nm) at a voltage of 40 kV and a current of 40 mA [24]. FTIR analysis (400–4000 cm^−1^) was used to determine the surface functional groups of CS-BC and MS-BC before and after adsorption. The ash content of the biochars was determined by combusting the samples in a muffle furnace at 873 K for 4 h and subsequent cooling in a desiccator until constant weight. Moreover, the zeta potential was determined at different pH values using a potential analyser (Zetasizer Nano, UK), while the pH of the biochar samples was measured by adding biochar to ultrapure water at a mass : water ratio of 1 : 20.

### 2.3. Batch adsorption experiments

Batch-mode adsorption studies were conducted to investigate the effects of the biochar loading, pH and coexisting anions on the Pb(II) adsorption process. CS-BC and MS-BC were added to 10 ml polyethylene (PE) centrifuge tubes containing a Pb(II) solution with an initial concentration of 40 mg l^−1^ at varying solid mass : liquid volume ratios (i.e. 0.0, 0.02, 0.04, 0.08, 0.1, 0.2, 0.4, 0.6, 0.8 and 1.0 g l^−1^) and the resulting solutions were shaken at 150 r.p.m. and 298 K for 8 h in a vertical temperature oscillation incubator (ZQPL-200,Tianjin Lai Bo Terry instrument Equipment Co., Tianjin, China). The suspensions were filtered with a 0.45 µm polysulfone filter membrane. The concentration of Pb(II) remaining in the supernatant solution was measured by inductively coupled plasma mass spectrometry (ICP-MS, Agilent 7500, USA) with a good method recovery (98 ± 8.7%) and a low detected limit (2 ng l^−1^). The optimal loading was selected for further experiments. To investigate the influence of the pH, the initial pH of the Pb(II) solutions was previously varied from 2 to 11 by using 0.1 mol l^−1^ NaOH or HNO_3_ solutions before performing the adsorption experiments as indicated above. The effect of coexisting anions (i.e. ${\mathrm{NO}}_{3}^{-}$, ${\mathrm{CO}}_{3}^{2-}$ and ${\mathrm{PO}}_{4}^{3-}$) on the adsorption of aqueous solutions of Pb(II) over biochar was studied by adding NaNO_3_, Na_2_CO_3_ and Na_3_PO_4_ at varying concentrations (0.001–0.1 mol l^−1^) in the initial Pb(II) solution. Every batch experiment was set with three parallel samples, and a blank solution experiment was conducted under the same test procedure. The removal efficiency and the adsorbed amount of Pb(II) ion at equilibrium were calculated using equations (2.1) and (2.2), respectively [25]:

2.1 $$\text{re}\mathrm{moval}\;(\%)=\frac{\left(C_{0}-C_{e}\right)}{C_{0}}\times 100\%$$

and

2.2 $$q_{e}(\mathrm{mg}\;g^{-1})=(C_{0}-C_{e})\times \frac{V}{m},$$

where *C*_0_ is the initial concentration of Pb(II) (mg l^−1^); *C_e_* represents the equilibrium concentration of Pb(II) (mg l^−1^); *q_e_* is the amount of adsorbed Pb(II) (mg g^−1^); *V* is the volume of Pb(II) solution (l) and *m* is the weight of CS-BC or MS-BC (g).

### 2.4. Adsorption kinetics

The kinetics for the adsorption of metal ions can be used to identify the main adsorption mechanism. Based on the above experiments, the optimal biochar loading, pH and anions concentration were selected for performing the adsorption kinetics, while the initial concentration of Pb(II) was fixed to 40 mg l^−1^. Liquid samples were collected at varying times (10, 20, 30 min and 1, 2, 3, 6 and 8 h) and the concentration of Pb(II) determined. In this study, pseudo-first-order and pseudo-second-order kinetic models were used to describe the adsorption process of Pb(II) ion adsorption on CS-BC and MS-BC. The pseudo-first-order and pseudo-second-order models can be expressed by the following equations [26]:

2.3 $$\log(q_{e}-q_{t})=\log q_{e}-\frac{K_{1}}{2.303}t\;(\text{pseudo-first-order})$$

and

2.4 $$\frac{t}{q_{t}}=\frac{1}{K_{2}\;{q_{e}}^{2}}+\frac{t}{q_{e}}\;(\text{pseudo-second-order}),$$

where, *q_t_* and *q_e_* (mg g^−1^) are the amounts of metal ions adsorbed at contact time *t* (min) and at equilibrium, respectively; *K*_1_ is the rate constant of the pseudo-first-order adsorption model (min^−1^); and *K*_2_ is the rate constant of the pseudo-second-order adsorption model (g mg^−1^ min^−1^).

### 2.5. Adsorption isotherm

For the adsorption isotherm study, Pb(II) aqueous solutions with varying initial concentrations (i.e. 20, 40, 60, 80, 100, 120, 140, 160, 180 and 200 mg l^−1^) were used at optimal pH, biochar loading, coexisting anion concentration and equilibrium time conditions previously determined. Furthermore, in order to study the effect of the temperature on the adsorption of Pb(II), adsorption experiments were conducted at 298, 313 and 328 K. Freundlich and Langmuir adsorption isotherm models were employed to fit the Pb(II) adsorption data on biochar. Langmuir and Freundlich isotherm models are, respectively, described as follows [27]:

2.5 $$q_{e}=\frac{Q_{\max}bC_{e}}{(1+b)C_{e}}\;(\mathrm{Langmuir})$$

and

2.6 $$q_{e}=K_{F}C_{e}^{n}\;(\mathrm{Freundlich}),$$

where *Q*_max_ is the maximum adsorption capacity of the biochar sample (mg g^−1^); *q_e_* is the equilibrium adsorption capacity of the biochar sample (mg g^−1^); *b* is the Langmuir adsorption characteristic constant (L mg^−1^); *K*_F_ and *n* represent the Freundlich empirical constants (l g^−1^) and *C*_e_ is the adsorption equilibrium concentration (mg l^−1^).

## 3. Results and discussion

### 3.1. Characterization of the biochar

The physico-chemical properties of CS-BC and MS-BC (i.e. productivity, ash content, pH and elemental composition) are listed in table 1. CS-BC showed a yield (44.4%) significantly higher than that of MS-BC (26.3%). The ash content of CS-BC (9.24%) was notably lower than that of MS-BC (48.7%) mainly because of the decomposition of volatile substances (CO_2_) and accumulation of minerals (KHCO_3_) at high content in the latter sample [9]. MS-BC showed higher BET surface area and MV as compared to CS-BC (table 1). There are the higher cellulose and lignin contents of corn straw than those of municipal sludge. High lignin-content biomass is not easily decomposed at low pyrolysis temperatures (less than 798 K), thereby resulting in incompletely developed pore structures. CS-BC and MS-BC were both weakly alkaline (average pH values of 8.18 and 7.36, respectively). CS-BC and MS-BC also showed low pH_pzc_ values (less than 5.0), thereby revealing high acidic characteristics and thus strong buffer capacity under basic environments [28]. The carbon content of CS-BC was significantly higher than that of MS-BC, which is consistent with the higher carbon content reported for other agricultural source-derived biochars [3,29]. The O/C and H/C ratios can be uses as an indication of the hydrophilicity and carbonization degree of biochar, respectively [30]. The higher value of O/C and H/C further confirmed that these biochars were incompletely decomposed under this pyrolysis temperature. Compared with CS-BC, MS-BC showed higher hydrophobicity and carbonization degree, thereby revealing a higher number of surface adsorption sites available for this biochar [3].

**Table 1.** Chemical and physical properties of the biochar samples.

	biochars	
characteristics, units	CS-BC	MS-BC
productivity,%	44.4	26.3
ash content,%	9.24	48.7
BET surface area, m^2^ g^−1^	4.36	10.1
MV, cm^3^ g^−1^	0.02	0.03
pH	8.18	7.36
pH_pzc_	4.05	2.08
C, %	64.5	31.6
H, %	3.83	2.10
O, %	19.9	20.2
N, %	2.67	5.49
S, %	0.39	0.28
O/C ratio	0.23	0.48
H/C ratio	0.71	0.80

**Figure 1.**
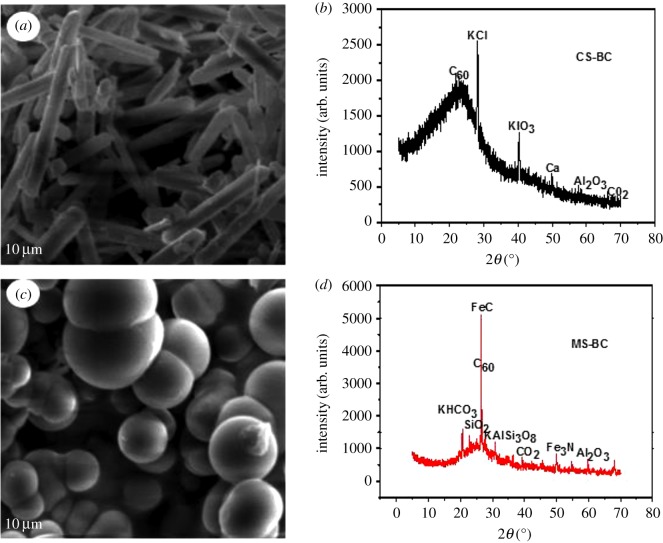
SEM images of (*a*) CS-BC and (*c*) MS-BC. XRD patterns of (*b*) CS-BC and (*d*) MS-BC.

SEM micrographs and XRD patterns of CS-BC and MS-BC are shown in figure 1. As shown in figure 1*a*,*c*, CS-BC showed a coarse fibre surface structure (4.36 m^2^ g^−1^), thereby revealing a significantly higher fraction of hemicellulose and cellulose as compared to MS-BC that showed a more smooth and slightly crumby structure with higher surface area (10.1 m^2^ g^−1^). Compared with CS-BC, some bright zones or points were clearly observed on the surface of MS-BC revealing the presence of Fe, Al and Si as determined by XRD analysis (figure 1*d*). These results were consistent with the findings of Singh *et al*. [31], who reported the presence of quartz, kaolinite, haematite and sylvite in cow manure- and poultry litter-derived biochars pyrolysed at 823 K. These minerals can co-precipitate along with heavy metal ions, thereby enhancing metal adsorption [3]. In the case of CS-BC, higher amounts of K^+^ and Ca^2+^ were observed (figure 1*b*), thereby allowing Pb(II) ions to be adsorbed on the biochar via electrostatic cation exchange with these ions or metal exchange reactions (i.e. surface complexes or precipitation) [32].

### 3.2. Effect of the biochar loading

**Figure 2.**
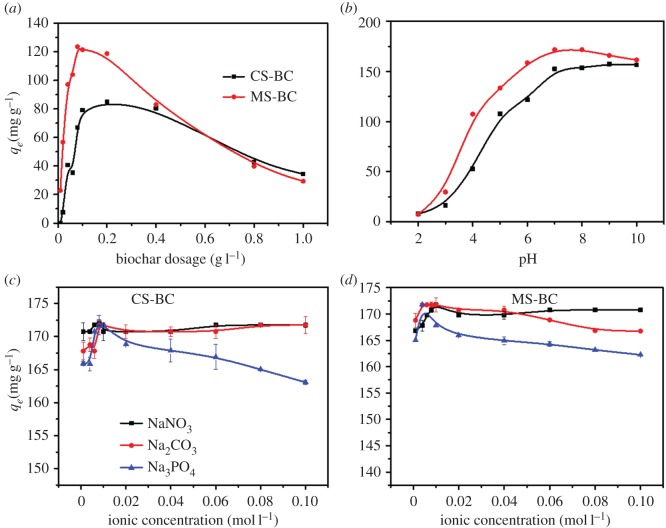
Effect of several parameters on the adsorption of Pb(II) on CS-BC and MS-BC: (*a*) biochar loading; (*b*) pH; (*c*) NaNO_3_, Na_2_CO_3_ and Na_3_PO_4_ concentrations for CS-BC and (*d*) NaNO_3_, Na_2_CO_3_ and Na_3_PO_4_ concentrations for MS-BC.

The biochar loading is an important factor during the adsorption process. As shown in figure 2*a*, both biochars showed similar change trends (i.e. the Pb(II) adsorption capacity increased with the biochar loading). In the case of MS-BC, the Pb(II) adsorption capacity rapidly increased up to 123 ± 0.21 mg g^−1^ with the dosage increasing from 0.0 to 0.1 g l^−1^ and slowly decreased thereafter (up to a biochar loading of 1.0 g l^−1^). In the case of CS-BC, the same trend was observed for biochar loadings of 0.0–0.2 g l^−1^ (largest Pb(II) adsorption capacity value of 84.8 ± 0.30 mg g^−1^). Although larger biochar loadings can provide more active sites for adsorption, the adsorption capacity of the biochar was observed to decrease at loadings above a certain value. At these conditions, biochar aggregation occurs thereby reducing the number of binding sites while also favouring electrostatic repulsion between the biochar and the metal ions [24]. The Pb(II) adsorption capacity over MS-BC was significantly higher than that of CS-BC because of the higher porosity and the presence of surface functional groups to a significantly larger extent in the former. CS-BC and MS-BC both showed their maximum Pb(II) adsorption capacity at a biochar loading of 0.2 g l^−1^. Thus, this biochar loading was selected herein as the optimum as it allows minimum utilization of the biochar samples, full use of their adsorption capability, and reasonable comparison between the adsorption behaviour of MS-BC and CS-BC.

### 3.3. Effect of pH

In natural aquatic system, Pb is present mainly as Pb^2+^ at pH < 6, Pb(OH)^+^, Pb(OH)_2_ at pH = 6–12 and ${\mathrm{Pb}(\mathrm{OH})}_{4}^{2-}$ at pH > 12, and Pb(OH)_2_ starts to form when pH exceeds 7.7 [24]. Thus, pH can affect the adsorption of Pb in aqueous solutions. As shown in figure 2*b*, the Pb(II) adsorption on MS-BC and CS-BC was dependent on the initial pH value of the solution, with 7 being the optimal pH conditions. The Pb(II) adsorption capacity on MS-BC increased up to 172 ± 0.3 mg g^−1^ with the pH varying in the range of 2–7 and slightly decreased thereafter. In the case of CS-BC, no adsorption of Pb(II) was observed at low pH values (2–4). The amount of Pb(II) adsorbed increased up to 157 ± 0.4 mg g^−1^ with the pH varying in the 4–7 range, remained constant thereafter (pH = 7–10), and decreased at pH values higher than 10. At low pH values, MS-BC (pH_pzc_ = 2.08) and CS-BC (pH_pzc_ = 4.05) were positively charged on the surface, and high electrostatic repelling forces inhibited the contact of Pb(II) ions and the biochar surface. At pH values higher than pH_pzc_, the electrostatic attraction between the biochar surface and the metal ions is enhanced because the biochar is negatively charged on its surface. Moreover, the concentration of H^+^ ions in solution and thus their competition with Pb(II) for surface adsorption sites decreases with the pH, thereby positively affecting the adsorption capacity of Pb(II). However, at higher pH values (pH > 6), Pb(II) can precipitate upon reaction with the OH^−^ ions, thereby reducing its mobility and leading to a lower adsorption capacity of MS-BC. The Pb(II) adsorption capacity of CS-BC remained constant in the pH range of 7–10, and this may be attributed to the protonation–deprotonation of carboxyl and hydroxyl groups on the biochar in the pH region [28]. The electrostatic interactions probably play an important role in controlling the removal of Pb(II) ions over biochar from aqueous solutions at different pH values, especially in the case of MS-BC.

### 3.4. Effect of coexisting anions

The effect of coexisting anions including ${\mathrm{NO}}_{3}^{-}$, ${\mathrm{CO}}_{3}^{2-}$ and ${\mathrm{PO}}_{4}^{3-}$ on the adsorption of Pb(II) over CS-BC and MS-BC are shown in figure 2*c* and *d*. The presence of ${\mathrm{PO}}_{4}^{3-}$ significantly affected the Pb(II) adsorption on MS-BC and CS-BC, with the optimal anion strength of ${\mathrm{NO}}_{3}^{-}$, ${\mathrm{CO}}_{3}^{2-}$ and ${\mathrm{PO}}_{4}^{3-}$ being 0.01 mol l^−1^. The Pb(II) adsorption capacity increased with the coexisting anion concentration increasing in the range of 0.0–0.01 mol l^−1^. When each coexisting anion concentration exceeded 0.01 mol l^−1^, different trends were observed for ${\mathrm{NO}}_{3}^{-}$, ${\mathrm{CO}}_{3}^{2-}$ and ${\mathrm{PO}}_{4}^{3-}$ on the adsorption capacity of Pb(II). In the case of CS-BC, the Pb(II) adsorption capacity remained constant at the maximum value (172 ± 0.03 mg g^−1^) while increasing the ${\mathrm{NO}}_{3}^{-}$ and ${\mathrm{CO}}_{3}^{2-}$ concentrations. In the case of MS-BC, the Pb(II) adsorption capacity levelled off (174 ± 0.06 mg g^−1^) with the concentration of ${\mathrm{NO}}_{3}^{-}$, whereas it slightly decreased upon increasing the ${\mathrm{CO}}_{3}^{2-}$ concentration. Increasing the ${\mathrm{PO}}_{4}^{3-}$ concentration above 0.01 mol l^−1^ resulted in a significant decrease of the Pb(II) adsorption capacity of both MS-BC and CS-BC (figure 2*b*). Thus, ${\mathrm{PO}}_{4}^{3-}$ inhibits the adsorption of Pb(II) on biochar above a certain concentration (0.01 mol l^−1^) because Pb(II) and ${\mathrm{PO}}_{4}^{3-}$ ions compete for the surface adsorption sites [11]. Additionally, when coexisting in solution, Pb(II) and ${\mathrm{PO}}_{4}^{3-}$ form Pb-phosphate precipitate (Pb_9_(PO_4_)_6_) that negatively affects the adsorption of Pb(II) on biochar [15].

### 3.5. Effect of the surface functional groups

**Figure 3.**
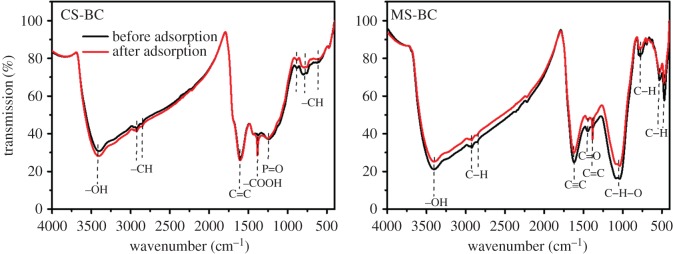
FTIR spectra of the biochar samples before and after adsorption of Pb(II).

The FTIR spectra of the two biochars were measured before and after Pb(II) adsorption (figure 3). As shown in figure 3, before Pb(II) adsorption both biochars exhibited absorption bands at 3407 cm^−1^ (─OH functional groups), 2919, 2852 and 796 cm^−1^ (─CH functional groups), 1611 cm^−1^ (C═C functional groups) and 1375 cm^−1^ (─COOH functional groups) [1,33]. Moreover, stretching P═O bands at 1242 cm^−1^ (CS-BC) and C═O bands at 1040 cm^−1^ (MS-BC) were observed. After Pb(II) adsorption, these bands corresponding to −OH, −CH and −COOH groups were notably displaced for both biochars, thereby indicating that the Pb(II) adsorption mechanisms were surface complexation (with carboxyl and hydroxyl functional groups) and coordination with the π electrons of aromatic ─CH groups [34,35]. In the case of MS-BC, other functional groups such as C═C and C═O showed noticeable vibrations, thereby suggesting that a significantly higher number of functional groups were involved in the Pb(II) removal via surface complexation and coordination. Similar results were reported for Pb(II) and Cd(II) adsorption on a sludge-derived biochar [14,24], thereby implying that the functional groups play an important role during the adsorption of heavy metals in aqueous solutions.

### 3.6. Adsorption kinetics

**Figure 4.**
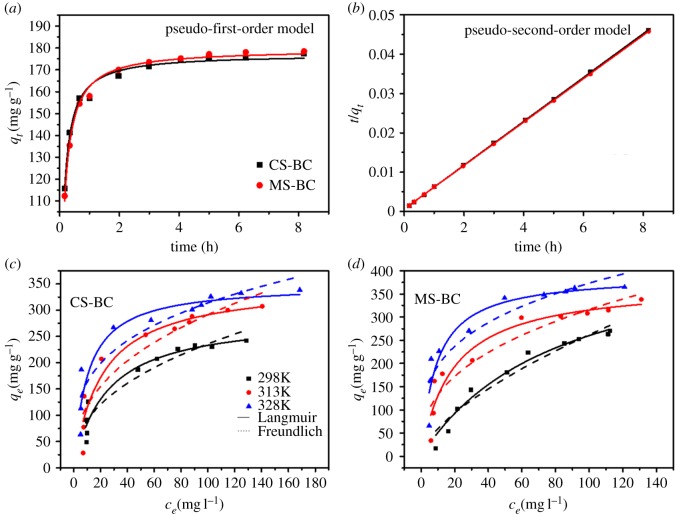
Adsorption kinetics (*a*,*b*) and adsorption isotherms (*c*,*d*) of Pb(II) on CS-BC and MS-BC.

Figure 4*a* and *b* shows the adsorption rate of Pb(II) on the two different biochars as well as the possible adsorption mechanism involved. The adsorption of Pb(II) on the biochar samples rapidly increased within the first 1 h and reached adsorption equilibrium after 8 h. Both biochars showed similar adsorption kinetics. Compared with CS-BC, MS-BC showed a higher Pb(II) adsorption capacity at equilibrium. Pseudo-first-order and pseudo-second-order kinetic models can be used to describe the adsorption of heavy metals [36]. The *K*_1_, *K*_2_, *q_e_* and *R*^2^ (correlation coefficient) values for these models are shown in table 2. As can be seen in table 2, unlike the pseudo-second-order kinetic model (*R*^2^ > 0.98), the correlation coefficients for the pseudo-first-order kinetic model were low (*R*^2^ < 0.80). Thus, a pseudo-second-order model was more suitable to describe the adsorption of Pb(II) on biochars, thereby revealing that chemical interactions between Pb(II) and the surface adsorption sites occurred during the adsorption process [35].

**Table 2.** Adsorption kinetics parameters of Pb(II) on CS-BC and MS-BC.

	pseudo-first-order model			pseudo-second-order model		
biochars	*q_e_* mg g^−1^	*K*_1·_min^−1^	*R*^2^	*q_e_* mg g^−1^	*K*_2_ g mg^−1^ min^−1^	*R*^2^
CS-BC	88.0	0.10	0.788	177	0.06	0.985
MS-BC	85.5	0.09	0.793	178	0.05	0.988

### 3.7. Adsorption isotherms and the effect of temperature

Figure 4*c* and *d* shows the adsorption isotherm of Pb(II) on the two different biochars at three different temperatures (i.e. 298, 313 and 328 K). In this study, the adsorption isotherms were obtained at different Pb(II) concentrations (20–200 mg l^−1^). The Langmuir and Freundlich models were used to fit the adsorption data, and the corresponding parameters are listed in table 3. The results showed that the Langmuir model provided a better fit to the Pb(II) adsorption on biochars as compared to the Freundlich model (*R*^2^: 0.89–0.95 versus 0.84–0.94), which further confirmed that chemisorption of Pb(II) is probably taking place on the surface of biochars during the Pb(II) adsorption process. The Pb(II) adsorption capacity on CS-BC and MS-BC significantly increased with the increase of initial Pb(II) concentration and the solution temperature. The maximum Pb(II) adsorption capacities of CS-BC and MS-BC obtained by the Langmuir model at 328 K were 352 and 387 mg/g for CS-BC and MS-BC, respectively. As confirmed by previous reports, the adsorption of Pb(II) on agricultural biochars is an endothermic process [33] and, therefore, the Pb(II) adsorption capacity increased with temperature.

**Table 3.** Parameters derived from the adsorption isotherm models and thermodynamic parameters for the Pb(II) adsorption on CS-BC and MS-BC.

		Langmuir model			Freundlich model			thermodynamic parameters		
		*Q*_max_	*b*					Δ*G*^0^	Δ*S*^0^	Δ*H*^0^
biochars	*T* (K)	mg g^−1^	l mg^−1^	*R*^2^	*K*_F_	*n*	*R*^2^	kJ mol^−1^	J mol^−1 ^K^−1^	kJ mol^−1^
CS-BC	298	288	0.04	0.931	27.1	0.47	0.911	−6.11	72.3	15.4
	313	350	0.05	0.923	45.2	0.43	0.880	−7.34		
	328	352	0.06	0.925	54.1	0.32	0.863	−8.27		
MS-BC	298	289	0.01	0.951	13.5	0.64	0.941	−6.28	283	80.8
	313	355	0.08	0.927	55.4	0.38	0.882	−8.81		
	328	387	0.17	0.894	102	0.28	0.844	−11.5		

To further evaluate the interactions between Pb(II) and biochars while varying the temperature, thermodynamic parameters such as the Gibbs free energy change (Δ*G*^0^), the standard enthalpy (Δ*H*^0^) and the standard entropy (Δ*S*^0^) were calculated using the following equations:

3.1 $$\begin{array}{rl} \Delta G^{0} & =-RT\;\ln\;K, \end{array}$$

3.2 $$\begin{array}{rl} \Delta G^{0} & =\Delta H^{0}-T\Delta S^{0} \end{array}$$

3.3 $$\begin{array}{rl} \;\mathrm{and}\;\ln\;K & =\frac{\Delta S^{0}}{R}-\frac{\Delta H^{0}}{RT}, \end{array}$$

where *R* is the gas constant (8.314 J K^−1^ mol^−1^), *T* is the absolute temperature in Kelvin and *K* is an equilibrium constant obtained by multiplying the Langmuir constants *Q*_max_ and *b*. The calculated thermodynamic parameters are listed in table 3. The negative values of Δ*G*^0^ suggested that the adsorption of Pb(II) on CS-BC and MS-BC was a thermodynamically favourable and spontaneous process. The positive value of Δ*S*^0^ revealed an increase in the disorder of the solid solution system [37]. The positive value of Δ*H*^0^ confirmed that the adsorption of Pb(II) on both biochars was an endothermic process. The high values obtained might account for the energy required to destroy the hydration sheath of Pb(II) with molecular water and to form some chemical bonds between Pb(II) and the functional groups in biochar during the adsorption process [37].

### 3.8. Assessment of the Pb(II) removal performance

Both CS-BC and MS-BC showed high Pb(II) removal capability in water solution at room temperature (table 4). Thus, both CS-BC and MS-BC can be potentially used as efficient adsorbents for removing heavy metals such as Pb(II) from aqueous solutions. The Pb(II) removal potential from aqueous solutions of the biochars tested herein (CS-BC: 288 mg g^−1^ and MS-BC: 289 mg g^−1^) was significantly higher than those of castor oil cake- (15.9 mg g^−1^) [38], wheat straw- (32 mg g^−1^) [32], sugar cane- (87 mg g^−1^), orange peel- (28 mg g^−1^) [3] and plum stone- (179 mg g^−1^) [32] derived biochars, and close to those of grape stalk- (273 mg g^−1^) [32] and *Alternanthera philoxeroides*- (257 mg g^−1^) [40] derived biochars. However, CS-BC and MS-BC showed the lower Pb(II) removal potential as compared to grape husk- (595 mg g^−1^) [24] and waste-art-paper- (1200 mg g^−1^ and 1500 mg g^−1^) [39] derived biochars. According to the highest adsorption capacity of Pb(II) calculated by the Langmuir isotherm model (table 2), the BET surface area of the biochars did not seem to be the key factor controlling the adsorption of Pb(II). Chemisorption might be the controlling mechanism during Pb(II) adsorption on these biochars. At room temperature, the Pb(II) removal performance in aqueous solutions was nearly similar for both CS-BC and MS-BC. However, considering the yield of the biochar, the productivity of corn straw (44.4%) was notably higher than that of municipal sewage (26.3%). Thus, CS-BC has a big potential for the removal of Pb(II) from aqueous environment based on an economical point of view.

**Table 4.** Pb(II) removal efficiency over different biochars.

feedstock	pyrolysis temperature (K)	productivity (%)	BET surface area (m^2^ g^−1^)	*Q*_max_^a^ (mg g^−1^)	reference
sugar cane bagasse	773	/	92.3	87.0	[3]
orange peel		/	0.21	27.9	
castor oil cake	673	/	1.10	15.9	[38]
wheat straw	873	18.9	364	31.8	[32]
grape stalk		30.6	72.0	273	
grape husk		31.6	77.0	595	
plum stone		24.7	443	179	
waste-art-paper	723	51.6	23.7	1200	[39]
	873	49.4	78.6	1500	
*Alternanthera philoxeroides*	873	/	19.8	257	[40]
corn straw	723	44.4	4.36	288	in the study
municipal sewage		26.3	10.1	289	

^a^*Q*_max_: the maximum Pb(II) adsorption capacities of biochar in the water solution at 298 K. It was calculated based on the Langmuir model.

## 4. Conclusion

CS-BC and MS-BC both showed good potential for the removal of Pb(II) from aqueous solutions. The adsorbents loading, pH, temperature of the solutions and the functional groups on the biochar significantly influenced the Pb(II) adsorption performance on these biochars. CS-BC and MS-BC showed a maximum Pb(II) adsorption capacity of 352 and 387 mg g^−1^, respectively, at pH = 7.0, 0.2 g l^−1^ biochar loading, 0.01 mol l^−1^ anion strength and 328 K. Unlike ${\mathrm{PO}}_{4}^{3-}$ coexisting ${\mathrm{NO}}_{3}^{-}$ and ${\mathrm{CO}}_{3}^{2-}$ in aqueous solutions did not significantly influence the adsorption of Pb(II). The adsorption experimental data were well fitted with Langmuir isotherm and pseudo-second-order kinetic models. Electrostatic interaction and surface complexation and coordination were suggested as Pb(II) adsorption mechanisms on biochars. The adsorption of Pb(II) over CS-BC and MS-BC was mainly carried out by chemisorption.

## Data accessibility

All data are provided in the main text.

## References

[1] Zhu Q, Wu J, Wang L, Yang G, Zhang X 2016 Adsorption characteristics of Pb2^+^ onto wine lees-derived biochar. Bull. Environ. Contam. Toxicol. 97, 294–299. (doi:10.1007/s00128-016-1760-4) 26920696 10.1007/s00128-016-1760-4

[2] Chaudhary MZ, Ahmad N, Mashiatullah A, Ahmad N, Ghaffar A 2013 Geochemical assessment of metal concentrations in sediment core of Korangi Creek along Karachi Coast, Pakistan. Environ. Monit. Assess. 185, 6677–6691. (doi:10.1007/s10661-012-3056-4) 23279880 10.1007/s10661-012-3056-4

[3] Abdelhafez AA, Li J 2016 Removal of Pb(II) from aqueous solution by using biochars derived from sugar cane bagasse and orange peel. J. Taiwan Inst. Chem. Eng. 61, 367–375. (doi:10.1016/j.jtice.2016.01.005)

[4] Huang Y, Wu D, Wang X, Huang W, Lawless D, Feng X 2016 Removal of heavy metals from water using polyvinylamine by polymer-enhanced ultrafiltration and flocculation. Sep. Purif. Technol. 158, 124–136. (doi:10.1016/j.seppur.2015.12.008)

[5] Papageorgiou SK, Katsaros FK, Kouvelos EP, Kanellopoulos NK 2009 Prediction of binary adsorption isotherms of Cu^2+^, Cd^2+^ and Pb^2+^ on calcium alginate beads from single adsorption data. J. Hazard. Mater. 162, 1347–1354. (doi:10.1016/j.jhazmat.2008.06.022) 18653278 10.1016/j.jhazmat.2008.06.022

[6] Sud D, Mahajan G, Kaur MP 2008 Agricultural waste material as potential adsorbent for sequestering heavy metal ions from aqueous solutions—a review. Bioresour. Technol. 99, 6017–6027. (doi:10.1016/j.biortech.2007.11.064) 18280151 10.1016/j.biortech.2007.11.064

[7] Yargıç AŞ, Yarbay Şahin RZ, Özbay N, Önal E 2015 Assessment of toxic copper(II) biosorption from aqueous solution by chemically-treated tomato waste. J. Clean. Prod. 88, 152–159. (doi:10.1016/j.jclepro.2014.05.087)

[8] Zhang R, Chen C, Li J, Wang X 2015 Preparation of montmorillonite@carbon composite and its application for U(VI) removal from aqueous solution. Appl. Surf. Sci. 349, 129–137. (doi:10.1016/j.apsusc.2015.04.222)

[9] Fan S, Tang J, Wang Y, Li H, Zhang H, Tang J, Wang Z, Li X 2016 Biochar prepared from co-pyrolysis of municipal sewage sludge and tea waste for the adsorption of methylene blue from aqueous solutions: kinetics, isotherm, thermodynamic and mechanism. J. Mol. Liq. 220, 432–441. (doi:10.1016/j.molliq.2016.04.107)

[10] Fornes F, Belda RM, Lidon A 2016 Analysis of two biochars and one hydrochar from different feedstock: focus set on environmental, nutritional and horticultural considerations. J. Clean. Prod. 86, 40–48. (doi:10.1016/j.jclepro.2014.08.057)

[11] Tan X, Liu Y, Zeng G, Wang X, Hu X, Gu Y, Yang Z 2015 Application of biochar for the removal of pollutants from aqueous solutions. Chemosphere 125, 70–85. (doi:10.1016/j.chemosphere.2014.12.058) 25618190 10.1016/j.chemosphere.2014.12.058

[12] Hegazi HA 2013 Removal of heavy metals from wastewater using agricultural and industrial wastes as adsorbents. HBRC J. 9, 276–282. (doi:10.1016/j.hbrcj.2013.08.004)

[13] Abdel-Fattah TM, Mahmoud ME, Ahmed SB, Huff MD, Lee JW, Kumar S 2015 Biochar from woody biomass for removing metal contaminants and carbon sequestration. J. Ind. Eng. Chem. 22, 103–109. (doi:10.1016/j.jiec.2014.06.030)

[14] Zuo WQ, Chen C, Cui HJ, Fu ML 2017 Enhanced removal of Cd(II) from aqueous solution using CaCO_3_ nanoparticle modified sewage sludge biochar. RSC Adv. 7, 16 238–16 243. (doi:10.1039/C7RA00324B)

[15] Bogusz A, Oleszczuk P, Dobrowolski R 2015 Application of laboratory prepared and commercially available biochars to adsorption of cadmium, copper and zinc ions from water. Bioresour. Technol. 196, 540–549. (doi:10.1016/j.biortech.2015.08.006) 26295440 10.1016/j.biortech.2015.08.006

[16] Higashikawa FS, Conz RF, Colzato M, Cerri CEP, Alleoni LRF 2015 Effects of feedstock type and slow pyrolysis temperature in the production of biochars on the removal of cadmium and nickel from water. J. Clean. Prod. 137, 965–972. (doi:10.1016/j.jclepro.2016.07.205)

[17] Liu J, Wu J, Liu F, Han X 2012 Quantitative assessment of bioenergy from crop stalk resources in inner mongolia, China. Appl. Energ. 93, 305–318. (doi:10.1016/j.apenergy.2011.12.059)

[18] Yang G, Zhang G, Wang H 2015 Current state of sludge production, management, treatment and disposal in China. Water Res. 78, 60–73. (doi:10.1016/j.watres.2015.04.002) 25912250 10.1016/j.watres.2015.04.002

[19] Chen YQ, Yang HP, Wang XH, Zhang SH, Chen HP 2012 Biomass-based pyrolytic polygeneration system on cotton stalk pyrolysis: influence of temperature. Bioresour. Technol. 107, 411–418. (doi:10.1016/j.biortech.2011.10.074) 22209443 10.1016/j.biortech.2011.10.074

[20] Guo W, Huo SL, Feng JL, Lu XF 2017 Adsorption of perfluorooctane sulfonate (PFOS) on corn straw-derived biochar prepared at different pyrolytic temperatures. J. Taiwan Inst. Chem. E 78, 265–271. (doi:10.1016/j.jtice.2017.06.013)

[21] Guo W, Ai YJ, Men B, Wang SJ 2017 Adsorption of phenanthrene and pyrene by biochar produced from the excess sludge: experimental studies and theoretical analysis. Int. J. Environ. Sci. Technol. 14, 1889–1896. (doi:10.1007/s13762-017-1272-8)

[22] USEPA. 1993 Standards for the use or disposal of sewage sludge. Fed. Regist. 58, 9248–9415.

[23] Qiu MY, Sun K, Jin J, Han LF, Sun HR, Zhao Y, Xia XH, Wu FC, Xing BS 2015 Metal/metalloid elements and polycyclic aromatic hydrocarbon in various biochars: the effect of feedstock, temperature, minerals, and properties. Environ. Pollut. 206, 298–305. (doi:10.1016/j.envpol.2015.07.026) 26219071 10.1016/j.envpol.2015.07.026

[24] Mohan D, Singh P, Sarswat A, Steele PH, Pittman CU Jr 2015 Lead sorptive removal using magnetic and nonmagnetic fast pyrolysis energy cane biochars. J. Colloid Interface Sci. 448, 238–250. (doi:10.1016/j.jcis.2014.12.030) 25744855 10.1016/j.jcis.2014.12.030

[25] Huang SH, Chen DH 2009 Rapid removal of heavy metal cations and anions from aqueous solutions by an amino-functionalized magnetic nano-adsorbent. J. Hazard. Mater. 163, 174–179. (doi:10.1016/j.jhazmat.2008.06.075) 18657903 10.1016/j.jhazmat.2008.06.075

[26] Yao Y, Gao B, Inyang M, Zimmerman AR, Cao X, Pullammanappallil P, Yang L 2011 Biochar derived from anaerobically digested sugar beet tailings: characterization and phosphate removal potential. Bioresour. Technol. 102, 6273–6278. (doi:10.1016/j.biortech.2011.03.006) 21450461 10.1016/j.biortech.2011.03.006

[27] Zama EF, Zhu YG, Reid BJ, Sun GX 2017 The role of biochar properties in influencing the sorption and desorption of Pb(II), Cd(II) and As(III) in aqueous solution. J. Clean. Prod. 148, 127–136. (doi:10.1016/j.jclepro.2017.01.125)

[28] Cui X, Fang S, Yao Y, Li T, Ni Q, Yang X, He Z 2016 Potential mechanisms of cadmium removal from aqueous solution by *Canna indica* derived biochar. Sci. Total Environ. 562, 517–525. (doi:10.1016/j.scitotenv.2016.03.248) 27107650 10.1016/j.scitotenv.2016.03.248

[29] Yuan H, Lu T, Huang H, Zhao D, Kobayashi N, Chen Y 2015 Influence of pyrolysis temperature on physical and chemical properties of biochar made from sewage sludge. J. Anal. Appl. Pyrol. 112, 284–289. (doi:10.1016/j.jaap.2015.01.010)

[30] Zhang G, Zhang Q, Sun K, Liu X, Zheng W, Zhao Y 2011 Sorption of simazine to corn straw biochars prepared at different pyrolytic temperatures. Environ. Pollut. 159, 2594–2601. (doi:10.1016/j.envpol.2011.06.012) 21719171 10.1016/j.envpol.2011.06.012

[31] Singh B et al. 2014 Opportunities and constraints for biochar technology in Australian agriculture: looking beyond carbon sequestration. Soil Res. 52, 739 (doi:10.1071/SR14112)

[32] Trakal L, Bingol D, Pohorely M, Hruska M, Komarek M 2014 Geochemical and spectroscopic investigations of Cd and Pb sorption mechanisms on contrasting biochars: engineering implications. Bioresour. Technol. 171, 442–451. (doi:10.1016/j.biortech.2014.08.108) 25226061 10.1016/j.biortech.2014.08.108

[33] Zhou G, Sun B, Zeng D, Wei H, Liu Z, Zhang B 2014 Vertical distribution of trace elements in the sediment cores from major rivers in east China and its implication on geochemical background and anthropogenic effects. J. Geochem. Explor. 139, 53–67. (doi:10.1016/j.gexplo.2013.03.007)

[34] Chen T, Zhou Z, Han R, Meng R, Wang H, Lu W 2015 Adsorption of cadmium by biochar derived from municipal sewage sludge: impact factors and adsorption mechanism. Chemosphere 134, 286–293. (doi:10.1016/j.chemosphere.2015.04.052) 25966459 10.1016/j.chemosphere.2015.04.052

[35] Lu H, Zhang W, Yang Y, Huang X, Wang S, Qiu R 2012 Relative distribution of Pb^2+^ sorption mechanisms by sludge-derived biochar. Water Res. 46, 854–862. (doi:10.1016/j.watres.2011.11.058) 22189294 10.1016/j.watres.2011.11.058

[36] Zhao J, He MC 2014 Theoretical study of heavy metal Cd, Cu, Hg, and Ni(II) adsorption on the kaolinite surface. Appl. Surf. Sci. 317, 718–723. (doi:10.1016/j.apsusc.2014.08.162)

[37] Sun J, Lian F, Liu Z, Zhu L, Song Z 2014 Biochars derived from various crop straws: characterization and Cd(II) removal potential. Ecotox. Environ. Safe 106, 226–231. (doi:10.1016/j.ecoenv.2014.04.042) 10.1016/j.ecoenv.2014.04.042 24859708

[38] Kalinke C, Mangrich AS, Marcolino-Junior LH, Bergamini MF 2016 Biochar prepared from castor oil cake at different temperatures: a voltammetric study applied for Pb^2+^, Cd^2+^ and Cu^2+^ ions preconcentration. J. Hazard. Mater. 318, 526–532. (doi:10.1016/j.jhazmat.2016.07.041) 27469040 10.1016/j.jhazmat.2016.07.041

[39] Xu X, Hu X, Ding Z, Chen Y, Gao B 2017 Waste-art-paper biochar as an effective sorbent for recovery of aqueous Pb(II) into value-added PbO nanoparticles. Chem. Eng. J. 308, 863–871. (doi:10.1016/j.cej.2016.09.122)

[40] Yang Y, Wei Z, Zhang X, Chen X, Yue D, Yin Q, Xiao L, Yang L 2014 Biochar from *Alternanthera philoxeroides* could remove Pb(II) efficiently. Bioresour. Technol. 171, 227–232. (doi:10.1016/j.biortech.2014.08.015) 25203230 10.1016/j.biortech.2014.08.015

